# Mesenchymal stem cells alleviate idiopathic pneumonia syndrome by modulating T cell function through CCR2-CCL2 axis

**DOI:** 10.1186/s13287-021-02459-7

**Published:** 2021-07-02

**Authors:** Min Cao, Huihui Liu, Yujun Dong, Wei Liu, Zhengyu Yu, Qingya Wang, Qingyun Wang, Zeying Liang, Yuan Li, Hanyun Ren

**Affiliations:** Department of Hematology, Peking University First Hospital, No. 8 Xishiku Street, Beijing, 100034 China

**Keywords:** Mesenchymal stem cell, Idiopathic pneumonia syndrome, T lymphocyte, Chemokine, CCR2-CCL2 axis

## Abstract

**Background:**

Idiopathic pneumonia syndrome (IPS) is a non-infectious fatal complication characterized by a massive infiltration of leukocytes in lungs and diffuse pulmonary injury after allogeneic hematopoietic stem cell transplantation (allo-HSCT). Conventional immunosuppressive treatments for IPS have poor therapeutic effects. Safe and effective treatments are not yet available and under explorations. Our previous study demonstrated that mesenchymal stem cells (MSCs) can alleviate IPS, but the mechanisms remain unclear.

**Methods:**

Co-cultured pre-activated T cells and MSCs in vitro to observe the changes in the CCR2-CCL2 axis. By establishing an IPS mouse model and administering MSCs to further verify the results of in vitro experiments.

**Results:**

Co-culture of pre-activated T cells with MSCs in vitro modulated the CCR2-CCL2 axis, resulting in quiescent T cells and polarization toward CCR2^+^CD4^+^ T cell subsets. Blocking CCR2-CCL2 interaction abolished the immunoregulatory effect of MSCs, leading to re-activation of T cells and partial reversion of polarizing toward CCR2^+^CD4^+^ T cells. In IPS mouse model, application of MSCs prolonged the survival and reduced the pathological damage and T cell infiltration into lung tissue. Activation of CCR2-CCL2 axis and production of CCR2^+^CD4^+^ T cells were observed in the lungs treated with MSCs. The prophylactic effect of MSCs on IPS was significantly attenuated by the administration of CCR2 or CCL2 antagonist in MSC-treated mice.

**Conclusions:**

We demonstrated an important role of CCR2-CCL2 axis in modulating T cell function which is one of the mechanisms of the prophylactic effect of MSCs on IPS.

**Supplementary Information:**

The online version contains supplementary material available at 10.1186/s13287-021-02459-7.

## Background

Idiopathic pneumonia syndrome (IPS) is one of the early fatal complications after allogeneic hematopoietic stem cell transplantation (allo-HSCT), with a high mortality rate and poor treatment response [1–3]. It is also considered as the pulmonary manifestation of acute graft-versus-host disease (aGVHD) due to the high co-occurrence of the two diseases [4–6]. It is pathologically characterized by interstitial-alveolar pneumonia and interstitial fibrosis, but without identifiable infections. High-dose systemic corticosteroids and supportive care are the current standard treatment and immunosuppressant is currently used to prevent and reduce the risk of IPS. Despite attempts to reduce risk factors, the overall death rate from IPS remains quite high. Once established, regardless of therapy, the overall mortality rate is 75% [7]. There is an unmet need to develop effective and safe prophylaxis or treatment for IPS.

Mesenchymal stem cells (MSCs) are multipotent adult stem cells that exist in many tissues such as bone marrow, adipose tissue, and umbilical cord blood [8]. MSCs have immune suppressive functions. They can inhibit the proliferation of T and B cells and the killing activity of NK cells and enhance Treg activity [9–13]. The immunosuppressive activity can be enhanced in the presence of inflammatory cytokines in the environment [14–16]. Joo et al. [17] reported that in a GVHD mouse model, intravenously infused MSCs first reached the lungs and then migrated to other organs. In IPS, when MSCs enter lungs, the high level of inflammatory cytokines in the lung tissue can enhance the immunosuppressive effect of MSCs. These findings suggest that MSCs might be used to prevent or treat IPS. Indeed, our previous research demonstrated that the use of MSCs can significantly prolong the survival and alleviate lung injury in a mouse model of IPS [18]. However, the mechanisms are not fully elucidated.

It is currently believed that the production of cytokines and chemokines by injured lung tissue which enables the recruitment of alloreactive donor T lymphocytes to the lungs plays a key role in the development of IPS. Previous studies provided some evidences that chemokine receptor 2 (CCR2) and its main ligand chemokine ligand 2 (CCL2) might be involved in this process. CCR2 and CCL2 play an important role in monocyte recruitment, T cell polarization, and immune response during inflammatory processes [19–21]. It was shown that CCR2 play an important role in the migration of T cells, monocytes, and macrophages to GVHD target organs including the lung [22, 23]. In a mouse allo-HSCT model, CCL2 was highly expressed in the liver, skin, lung, and intestine, and CCR2 was highly expressed in the liver and lung tissues [24]. High expression of CCR2-CCL2 is essential for the immune suppressive effect of MSCs. For example, CCL2-deficient MSCs fail to establish long-lasting contact with T cells and no longer ameliorate lupus symptoms [25]. The lack of CCL2 expression impaired MSCs’ suppression on B cells in lupus [26]. On the other hand, CCR2^+^ exosome released by MSCs suppresses macrophage functions and alleviates ischemia/reperfusion-induced renal injury [27]. MSCs recruit CCR2^+^ monocytes to suppress allergic airway inflammation [28]. In a preclinical randomized controlled trial, Carlumab, a humanized monoclonal antibody targeting CCL2, failed to benefit patients with idiopathic pulmonary fibrosis and was even terminated early due to a trend towards worsening lung function in one of the treatment arms [29].

The prophylactic and therapeutic mechanism of MSCs on IPS has not been fully elucidated. Our preliminary experiment found that co-culture of MSCs and pre-activated T cells significantly increased the level of CCL2 in the supernatant and the expression of CCR2 on T cells. In this study, we aimed to investigate the function and mechanism of CCR2-CCL2 axis for the prophylactic effect MSCs on IPS using in vitro and in vivo models.

## Materials and methods

### Mice

SPF grade healthy male C57BL/6 (H2Kb) mice (6–8 weeks old, weight of 20–25 g) and female Balb/c(H2Kd) mice (6–8 weeks old, weight of 20–25 g) were purchased from Beijing Vital River Laboratory Animal Technology Co., Ltd. Mice were housed and cared in the Experimental Animal Center, Peking University Health Sciences Center. All the experimental procedures were approved by the Animal Experiments Ethics Committee of Peking University First Hospital.

### Mesenchymal stem cells

BM-MSCs were derived from the bone marrow of 6–8-week-old C57BL/6J mice. Mice were sacrificed by carbon dioxide asphyxiation and bone marrow cells were collected from the femur and tibia. Isolated cells were cultured in RPMI1640 culture medium (Gibco, USA) supplemented with 10% FBS, 100μg/ml penicillin, and 100μg/ml streptomycin at 37 °C, 5% CO_2_ and 100% humidity. Non-adherent cells were removed after 72 h, and the medium was changed every 4–5 days. Passages were proceeded when the adherent cells approached 80% confluence. Cells over three passages were harvested and used for experiments.

### Co-culture of MSCs and pre-activated T cells

Spleen cells from C57BL/6 mice were sorted using the MojoSort Mouse CD3 T Cell Isolation Kit (Biolegend, USA) to obtain CD3+ T cells. T cells (3 × 10^6^/ml) were stimulated with anti-CD3/CD28 antibodies (2μg/ml each, PeproTech, USA) for 4 h. In the co-culture system (n=5), different amounts of MSCs were added to each group at the T:MSC ratios of 2:1, 5:1, 10:1, and 20:1. In the Transwell system (n=5), the same pre-treated T cells and MSC ratios were used. T cells were added to the upper chamber and MSCs were added to the lower chamber to avoid the direct contact between the two cell types. Samples were collected at 24 h, 48 h, and 72 h.

### Mixed lymphocyte reaction

Spleen cells from BALB/c mice were pre-treated with 50 μg/ml mitomycin C (Sigma, USA) for 30 min at 37 °C. After being washed three times by PBS, 0.8 × 10^6^ BALB/c spleen cells (stimulator) were mixed with 0.8 × 10^6^ C57BL/6 spleen cells (responder) in 250 μl in RPMI1640 culture medium containing 10% FBS, 100μg/ml penicillin, and 100μg/ml streptomycin. Different doses of MSC were added at the same time. The cells are co-cultured in ordinary 96-well plates. After co-culture for 72 h, cells were collected and tested for T cell activation.

### IPS mouse model construction and processing

#### Animal model construction

Recipient mice (BALB/c) underwent 8Gy of total-body γ-irradiation (0.8Gy/min). Six hours after irradiation, 0.2-ml cell mixture containing 1.0×10^7^ bone marrow cells and 2.0×10^7^ spleen cells from C57BL/6 mice were injected via tail vein. A dose of 5 mg/kg lipopolysaccharide (LPS, 055: B5) of *Escherichia coli* (Sigma) was also injected via tail vein at the same time. Mice were divided into four experimental groups (A, B, C, and D). (A) IPS group, (B) IPS + MSCs co-transplantation group (injection of 0.2 ml/per mouse of 2.5 × 10^6^/ml BM-MSCs via the tail vein), (C) IPS + MSCs + CCR2 antagonist group (2mg/kg CCR2 antagonist (RS102895, MedChemExpress) was administered daily by intraperitoneal injection for 2 weeks); and (D) IPS + MSCs + CCL2 antagonist group (100mg/kg CCL2 antagonist (Bindarit, MedChemExpress) was administered daily by intraperitoneal injection for 2 weeks). The concentrations of Bindarit and RS504393 used in this study were based on previous publications [30, 31]. Each group included 15 mice. Two same experiments were conducted in parallel. One was used to observe the survival and GVHD score, and the other was used for periodic sacrifice for related laboratory tests.

#### Evaluation of clinical GVHD

Clinical assessment of the severity of GVHD in recipient mice was performed on days 7 and 14 after transplantation according to the GVHD grading system previously published by Cooke and colleagues [32]. A clinical GVHD score was calculated by summation of the five criteria scores (ranging 0–10), including weight loss (1, 10–25%; 2, >25%), posture (1, hunching only at rest; 2, severe hunching impairs movement), activity (1, stationary>45% of the time; 2, stationary unless stimulated), fur texture (1, mild to moderate ruffling; 2, ruffling entire body), and skin integrity (1, scaling paws/tails; 2, obvious areas of denuded skin).

#### Histological research

Mice in each group were sacrificed on days 7, 14, and 21 after transplantation. Bronchial alveolar lavage fluid (BALF) 1 ml was collected and stored at − 20 °C. The lung tissues were fixed in 10% buffered formalin and embedded in paraffin blocks for subsequent hematoxylin and eosin staining. Histologic changes were graded and scored by a pathologist blinded to the clinical status of the mice. Four items were assessed as described previously [32]. To determine the infiltration of T cells in the lungs of IPS mice, immunohistochemical analysis was performed for staining with primary anti-CD3 antibody as well as the appropriate horseradish peroxidase-conjugated secondary antibody on the lung tissues of recipient mice at 14 days post-transplantation according to the manufacturer’s instructions.

### Flow cytometry

Lymphocytes from MSC and T cell co-culture system at 24 h, 48 h, and 72 h and MLRs at 72 h were washed with PBS. Or in IPS mouse model, mice were sacrificed on days 7, 14, and 21, and lungs were digested in RPMI-1640 medium containing collagenase XI (Sigma-Aldrich) and type IV bovine pancreatic DNase (Sigma-Aldrich) to obtain single-cell suspensions. Lymphocytes were isolated using Percoll (Living, Beijing, China). Flow cytometry was used to analyze activated status of T cells by staining with CD69 antibody and subsets by staining with relevant antibodies.

In in vitro co-culture system, all samples were divided into two parts for flow cytometry. One was stained with fluorescent antibodies CD3-FITC, CD4-PE-Cy7, CD8-BV510, CD69-PE, and CCR2-APC for detecting T cell activation and the CCR2 expression level, and the other was stained with CD3-APC, CD105-PE, CD44-PE-Cy7, CD90-FITC, and CCL2-Percp-Cy5.5 to detect the expression and cell source of CCL2. For CCL2 tube staining, cells were cultured with Brefeldin A (Biolegend, CA, USA) for 6 h. Cells are stained with surface markers and then fixed and permeated with the Intrasure Kit (BD Biosciences, NJ, USA) according to the manufacturer’s intracellular for staining instructions. Isotype IgG was used as a blank control. For proliferation assays, Cell Trace carboxyfluorescein succinimidyl ester (CFSE) staining was performed according to manual instruction. T cells were stained by CFSE and the proliferation was evaluated based on CFSE dilution (compared with undivided peak of naïve T cells).

### ELISA

Commercially available ELISA kit (Biolegend) was used following the manufacturers’ instructions to determine the concentration of CCL2 in the BALF and supernatant of MSC and T cell co-culture system.

### Statistical analyses

Data were processed using GraphPad Prism 8.0. The results are expressed as mean ± standard deviation. Independent sample t test was used for comparison of two groups, and one-way ANOVA (analysis of variance) was used for multiple group comparison. Use K-M curve for survival analysis and Log-rank test for difference analysis. p < 0.05 is considered statistically significant.

## Results

### MSCs inhibit T cell activation and proliferation

**Fig. 1:**
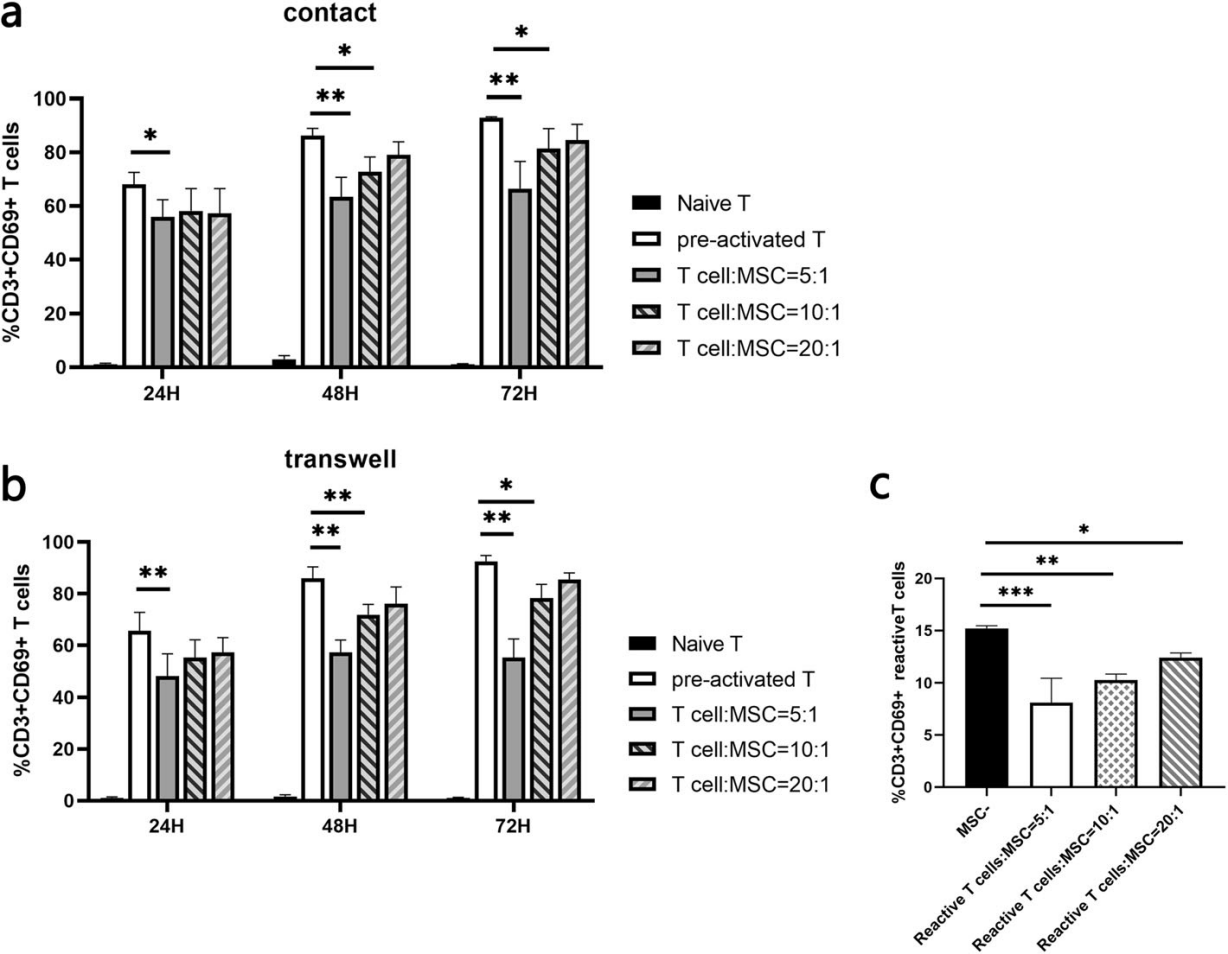
Effect of MSCs on T cell activation. T cells were pre-activated by adding CD3/CD28 monoclonal antibodies for 4 h and then co-cultured with MSCs for 72 h. T cells were collected at different time points to detect the effect of MSCs on T cell activation (**a**) in the contacting culture system or **b** by isolating MSCs and T cells in the Transwell system. **c** The inhibitory effect of MSCs on T cell activation in mixed lymphocyte reaction (MLR) when co-cultured with MSCs in the contacting culture system. T cell activation status was analyzed for detection of CD69-positive T cells using flow cytometry. *n* = 5, *P <0.05; **P <0.01; ***P <0.001

Figure 1 shows the inhibitory effect of MSCs on T cells at different time points. In the co-culture system, fewer CD3+CD69+ cells were observed in an MSC dose-dependent manner, indicating that inhibition of T cell activation is the main function of MSCs (Fig. 1a). And a dose-dependent inhibition effect of MSCs on T cell proliferation was also observed in the proliferation inhibition assay (Figure S1a). In the Transwell system, similar inhibitory effect on T cell activation and proliferation was observed, suggesting that direct contact with T cells is not required for MSCs to perform this function (Fig. 1b, Figure S1b). We conducted MLR experiments simulating the inflammatory environment in vivo in the contacting culture system and observed that MSCs had significant inhibitory effects on the activation and proliferation of T cells (Fig. 1c, Figure S1c).

### Co-culture of MSCs and pre-activated T cells changes CCL2-CCR2 axis

**Fig. 2:**
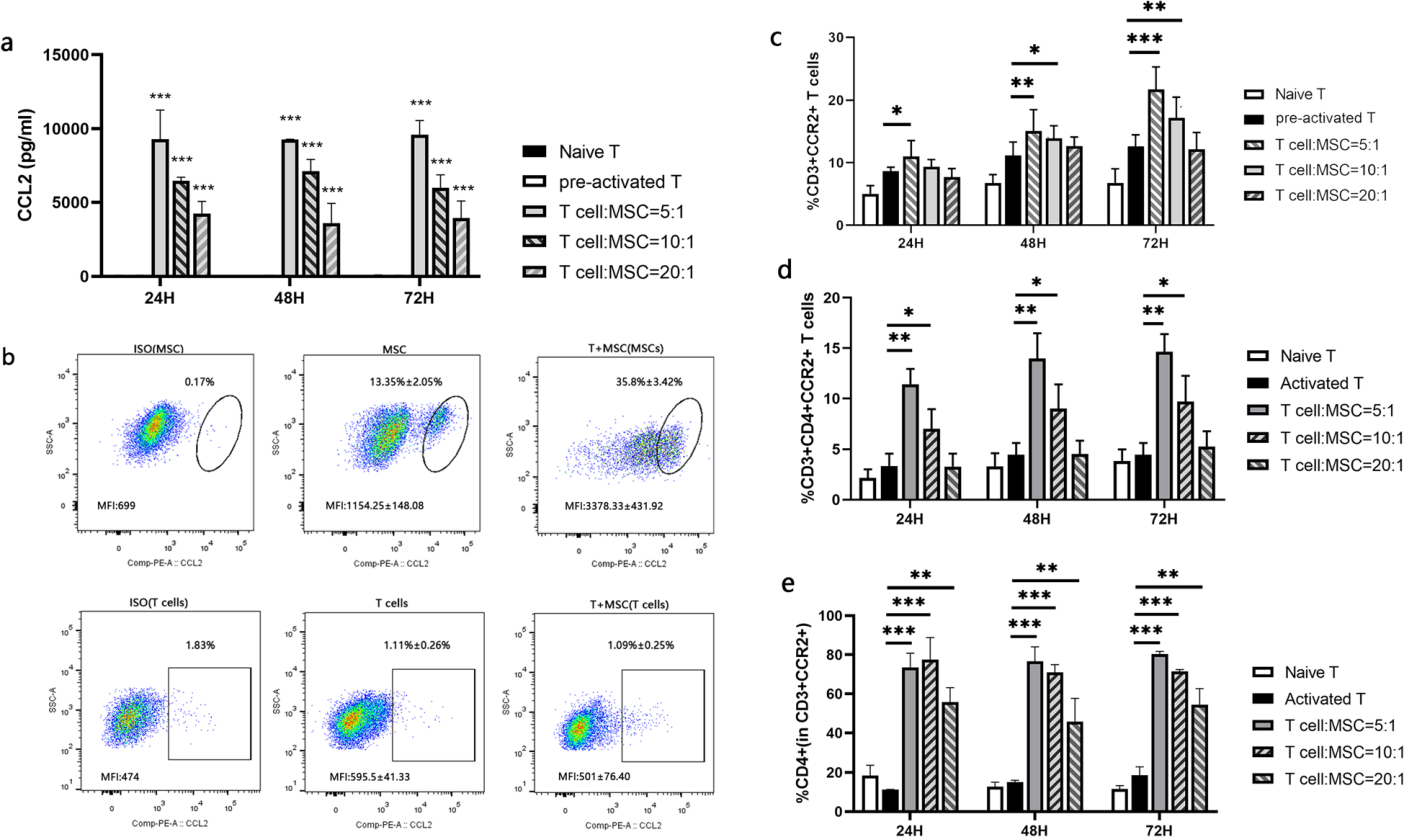
Effect of MSCs on CCR2-CCL2 axis in vitro. **a** The concentration of CCL2 in the supernatants of T cell culture only or with different ratio of MSCs detected using ELISA. **b** Flow cytometry analysis to detect the intracellular CCL2 in pre-activated T cells and MSCs in individual culture system and coculture system. **c** The percentage of CCR2+ T cells, **d** CCR2+CD4+ T cells and **e** CD4+ cells in CCR2+ T cells in co-culture system analyzed using flow cytometry at different time points. *n*=5, *P < 0.05; **P < 0.01; ***P < 0.001. Compared with the pre-activated T cells

After co-culture of MSCs with pre-activated T cells, the concentration of CCL2 in the supernatant was detected using ELISA. The production of CCL2 increased significantly after interaction of MSCs with pre-activated T cells (Fig. 2a). Flow cytometric analysis revealed that MSCs but not T cells are the source of increased CCL2 (Fig. 2b). We also found a significant increase in the proportion of CCR2-expressing T cells after co-culture with MSCs (Fig. 2c, d). Subset analysis showed that these CCR2-expressing T cells were mainly CCR2+CD4+ T cells. The proportion of CCR2+CD4+ T cells increased in an MSC dose-dependent manner (Fig. 2c, d). These results demonstrated that interaction of MSCs with pre-activated T cells increases the production of CCL2 by MSCs and polarization of T cells to CCR2+CD4+ T cells.

### Blocking interaction of CCL2 and CCR2 attenuates the inhibitory effect of MSCs on T cell activation and proliferation in vitro

**Fig. 3:**
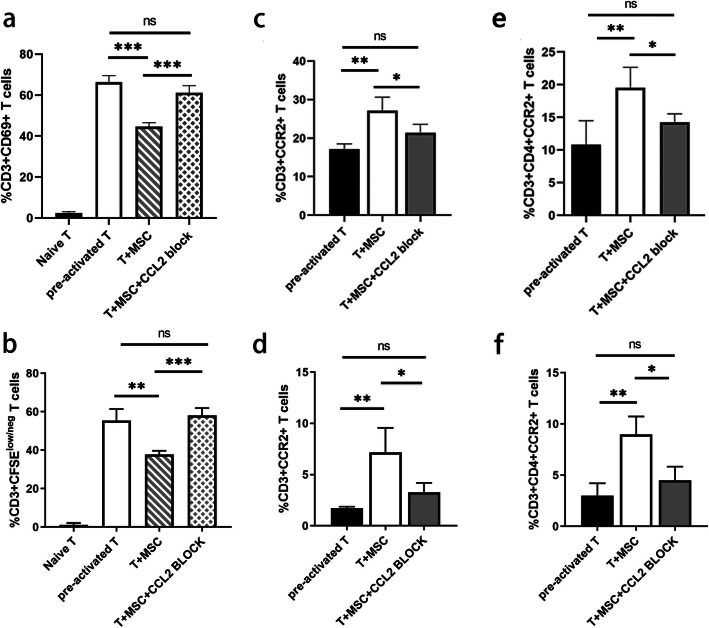
Blocking CCR2-CCL2 interaction lead to reverse the polarization of T cells toward CCR2+CD4+ T cells in vitro. The CCL2 neutralizing antibody was added to block CCL2 interaction with CCR2 in the co-culture system. Flow cytometry was used to detect **a** T cell activation, **b** T cell proliferation, **c** CCR2 expression on T cells in contacting culture system and **d** transwell system, **e** the proportion of CCR2+CD4+ T cells in contacting culture system and **f** transwell system. *n* = 5, *P <0.05; **P <0.01; ***P <0.001

In order to understand the role of CCL2 and CCR2 in the interaction of MSCs and T cells, we added CCL2 neutralizing antibody to the co-culture system. The inhibitory effect of MSCs on T cell activation and proliferation were significantly abolished (Fig. 3a, b), so were the effect on the increase in the proportion of CCR2+ T cells and CCR2+CD4+ T cells both in the contacting culture system (Fig. 3c, e) and the transwell system (Fig. 3d, f). These results demonstrated that inhibitory effect of MSCs on T cell activation and polarization of T cells toward CCR2+CD4+ T cells in vitro were achieved through the interaction of CCL2 and CCR2. And this effect is cell contact-independent.

### MSCs alleviate GVHD and lung pathological damage in IPS model

**Fig. 4:**
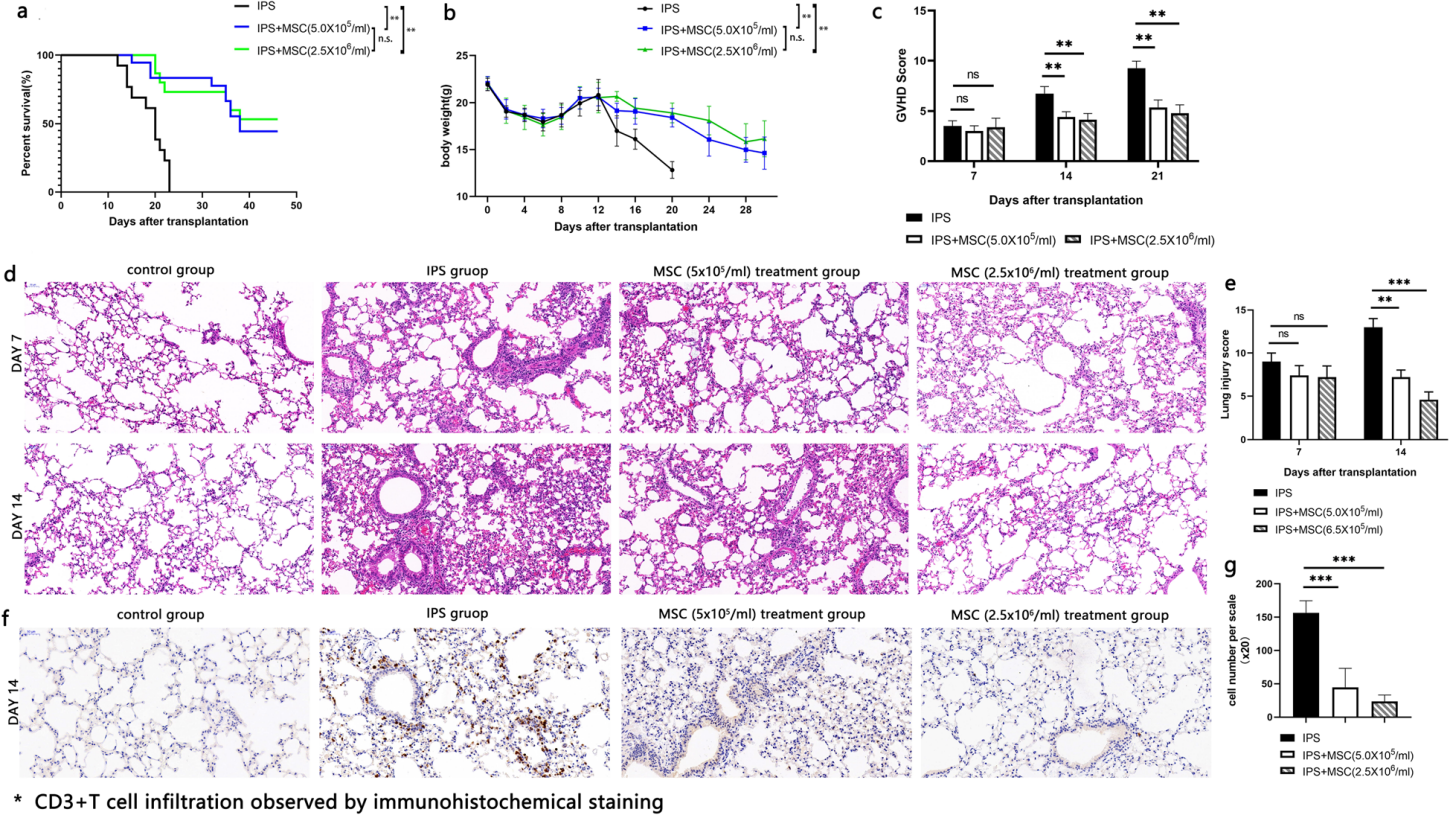
Prophylactic effect of MSCs on GVHD and lung pathological injury in IPS mice. MSCs were co-injected with bone marrow cells and spleen cells via the tail vein for prophylaxis of GVHD and lung injury in IPS mouse model. The effect of MSCs on **a** mouse survival, **b** weight changes, and **c** clinical scores of GVHD were shown. Mouse lung tissue specimens were collected for HE staining. **d** The pathological score of each group. **e** One of the representatives of pathological results in each group. **f** One of the representative results of CD3+ T cell infiltration observed by immunohistochemical staining. *n*=9, *P < 0.05; **P < 0.01; ***P < 0.001; n.s., no statistical difference

We established a mouse model of IPS to investigate the effect of MSCs in vivo. In this IPS model, all the mice manifested as apparent characteristics of aGVHD and died within 22 days after transplantation. Histopathological analysis showed that the lung tissue suffered a varied degree of alveolus collapse, lung edema, alveolar hemorrhage, inflammatory cell infiltration, and alveolar wall thickening (Fig. 3e, f). Prophylactic use of MSCs intravenously in this IPS model significantly prolonged the survival (Fig. 4a), reduced the weight loss (Fig. 4b), lowered GVHD clinical score (Fig. 4c), and alleviated the pathologic damage and T cell infiltration of the lung tissue (Fig. 4d–f). Different doses of MSCs (5 × 10^5^ or 2.5 × 10^6^/ml, 200 μl/mouse) for IPS prophylaxis showed almost equal effect on survival (Fig. 4a) and alleviating lung tissue injury (Fig. 4d–f). So we chose the dose of 2.5 × 10^6^/ml and 200 μl per mouse for subsequent experiments. These results confirmed that in vivo use of MSCs can alleviate GVHD and lung pathological damage in IPS mice.

### MSCs increase CCL2 level in BALF and the ratio of CCR2+CD4+ T cells in the lung tissue in IPS model

**Fig. 5:**
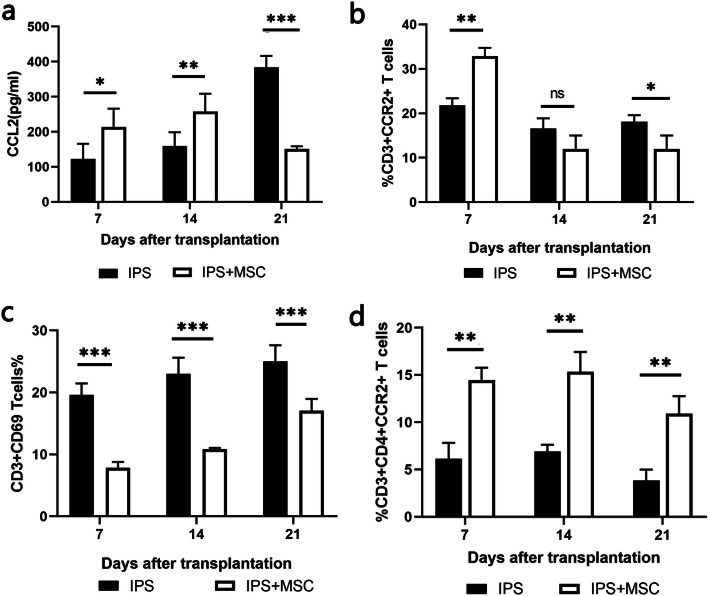
MSCs regulate CCR2-CCL2 axis in the lungs of IPS mice. The BALF was taken at different time points after transplantation. **a** The concentration of CCL2 detected by ELISA. **b** The percentage of CCR2 expressing lung-infiltrated T cells by flow cytometry. **c** The activation of T cells in the MSC-treated mice. **d** the proportion of CCR2+CD4+ T cells in the lungs of the MSC-treated mice and IPS mice. *n*=9, *P < 0.05; **P < 0.01; ***P < 0.001; n.s., no statistical difference

To validate the effect of MSCs in vivo, we examined the CCL2 content in BALF and CCR2+ T cells in lung tissue in IPS mouse model after intravenously use of MSCs on days 7, 14, and 21. ELISA showed that the concentration of CCL2 increased in BALF on days 7 and 14 in the MSC-treated mice than that in the IPS mice (*p* < 0.05). However, CCL2 declined thereafter in the MSC-treated group but continue to rise in the IPS group (Fig. 5a). The CCR2+ T cells in lung tissue in MSC-treated mice increased significantly than that in IPS mice on day 7 and started to decline from day 14 and reached a lower proportion than IPS group on day 21 (Fig. 5b). Meanwhile, fewer CD69+ T cells were found in the MSC-treated mice, indicating that T cell activation was inhibited in vivo after MSC infusion (Fig. 5c). T cell subset analysis showed that the proportion of CCR2+CD4+ T cells in the lung tissue was consistently higher in the MSC-treated mice (Fig. 5d).

### CCL2/CCR2 antagonists significantly weaken the prophylactic effect of MSCs on IPS

**Fig. 6:**
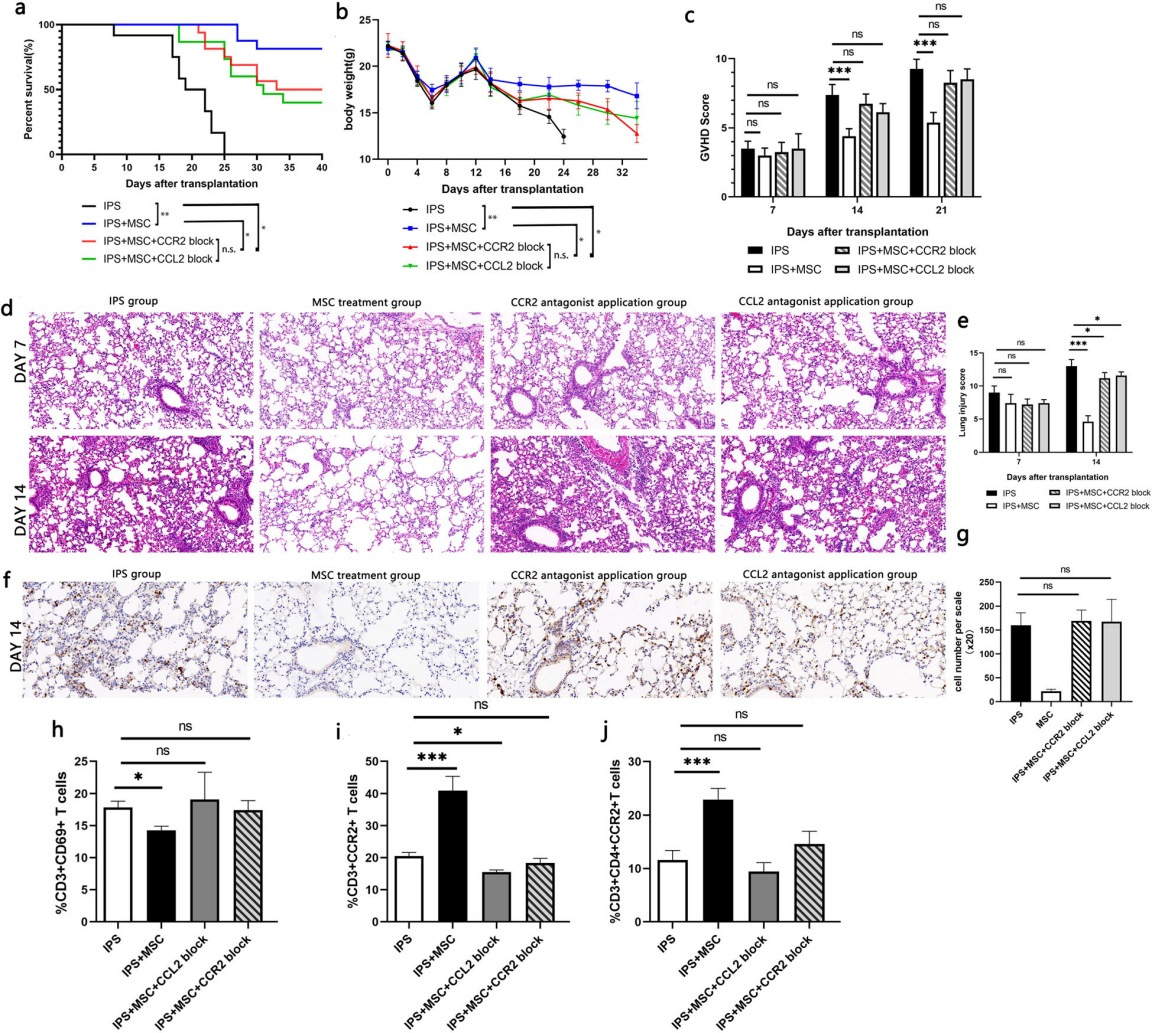
Application of CCR2/CCL2 antagonists impair the prophylactic effect of MSCs on IPS. In the IPS mouse model, CCL2 or CCR2 antagonist was used to block the interaction of CCL2 with CCR2 in vivo to observe the effect of MSCs on **a** survival rate, **b** weight changes, **c** clinical scores of GVHD, and **d** lung injury. **e** The representative pathological results of lung injury related to different conditions. **f** The representative pathological result of CD3+ T cell infiltration observed after immunohistochemical staining. **g** CD69+ T cells, **h** CCR2-expressing T cells, and **i** CCR2+CD4+ T cells in T cells isolated from lung tissue. *n*=9, *P < 0.05; **P < 0.01; ***P < 0.001. n.s., no statistical difference

To further validate the role of CCR2-CCL2 axis on IPS prophylactic effect of MSC infusion, MSC-treated mice were additionally administered CCR2 or CCL2 antagonists. The result showed that both antagonists significantly weakened the protective effect of MSCs, manifesting shortened survival (Fig. 6a), aggravated weight loss (Fig. 6b), deteriorated GVHD score (Fig. 6c), exacerbated lung tissue damage (Fig. 6d, e), and increased infiltration of T cells (Fig. 6f, g). In the further detection of the infiltrated T cell subsets in the lung tissue, the proportion of CCR2-expressing T cells especially CCR2+CD4+ T cells was found decreased (Fig. 6h–i) with enhanced re-activation of T cells after the application of the blockers.

## Discussion

IPS is a major complication after allogeneic hematopoietic stem cell transplantation (allo-HSCT) and involves the infiltration of donor leukocytes and the secretion of inflammatory cytokines and was also viewed as lung manifestation of GVHD. In this study, we found MSCs can alleviate symptoms in a mouse IPS model. We found that interaction of MSCs with pre-activated T cells led to significantly change the CCR2-CCL2 axis and suppressed T cell function in vitro. Using a mouse IPS model, we found that infusion of MSCs intravenously could also change the CCR2-CCL2 axis and decrease infiltration of T cells into lung tissue and alleviate lung injury. Further analysis showed that treatment with MSCs polarized T cells toward CCR2+CD4+ T cells both in vitro and in vivo. Using neutralizing antibody of CCL2 and antagonist of either CCR2 or CCL2 can abolish the prophylactic effect of MSCs.

Chemokines and their cognate receptors may play important roles in the pathogenesis of this complication. CCL2, also called monocyte chemoattractant protein-1 (MCP-1), is a major chemoattractant for several cell types, including macrophages and fibrocytes, but most strongly attracts T cells [33]. Early study in a mouse transplant model by Hildebrandt GC showed a critical role for CCR2/MCP-1 interactions in the development of IPS and patients with IPS have elevated levels of MCP-1 in the BAL fluid at the time of diagnosis [34]. However, study by Gurczynski et al. showed that loss of CCR2 signaling altered leukocyte recruitment and exacerbated herpesvirus-induced pneumonitis following allo-HSCT [35]. Some studies have showed that the immune regulatory effect of MSCs on a variety of inflammations rely on the effective CCL2/CCR2 signaling [26–29]. However, the mechanisms underlying the effect of MSCs on the prevention and treatment of IPS are still unclear.

In order to investigate the mechanisms underlying the prophylaxis of IPS with MSCs, we first co-cultured MSCs with pre-activated T cells in vitro, then analyze the concentration of CCL2 in the supernatant and the source of CCL2 in MSCs and T cells, and also analyze the activation status of T cells and CCR2 expression on T cells. The results showed that there is sharp increase in the secretion of CCL2 by MSCs and upregulation of the expression of CCR2 on T cells, and these effects were both MSC-dose dependent. These CCR2-expressed cells are mainly CD4+ T cells which account for more than 70% when T cell to MSC was 5:1 to 10:1 at 72-h culture. With the change of CCR2-CCL2 axis, more pre-activated T cells back to quiescent status. Blocking CCR2-CCL2 interaction using neutralizing antibody against CCL2 can significantly abolish this immunoregulatory effect of MSCs, leading to re-activation of T cells and partially reverse the polarization to CCR2+CD4+ T subset. In mouse IPS model, CCL2 content in BALF of mice treated with MSCs increased significantly, and the proportion of CCR2-expressing T cells especially CCR2+CD4+ T subset in the lung tissue also increased, which accompanied with alleviating lung pathological damage and T cell infiltration and significantly prolonged the survival. Both CCR2 and CCL2 antagonists can significantly reversed these effects of MSCs in vivo. These results demonstrated that the production of CCR2+CD4+ T cells might be the main mechanism underlying MSC-induced inhibitory effect on T cell immunity.

Recently, Milger et al. [36] first described a novel CCR2+CD4+ T cell subset from lung tissue of idiopathic pulmonary fibrosis (IPF), and the frequencies of this T cell subset were increased in experimental fibrosis. Importantly, adoptive transfer of CCR2+CD4+ T cells attenuated inflammation and fibrosis development, and this immunoregulatory effect was confirmed by in vitro suppressor assays. Further study showed that a higher proportion of FOXP3+ subset in CCR2+CD4+ T cells. These results indicated that CCR2+CD4+ T cells are immunoregulatory cells capable of suppressing lung inflammation and fibrosis [36]. This phenomenon is similar to our result which demonstrated that CCR2+CD4+ T cells possess immunoregulatory character and play important roles in attenuating inflammation and lung injury in IPS.

In vivo study showed concentration of CCL2 in BALF was increased in the MSC-treated mice mainly at the early days, consistent to the previous studies which have shown that MSCs accumulate in the lungs in the early stage of transplantation [37], and that MSCs are the main source for CCL2. CCL2 in late stage may come from lung interstitial cells, which can also secrete CCL2 [38]. CCL2 plays an important role in T cell recruitment and T cell apoptosis. A study found that MSCs attracted activated T cells through the CCL2 pathway and showed long-term inhibitory effect on T cells, and MSCs lacking CCL2 would lose this function [26]. This may explain our observations that although CCL2 increased only at the early days after transplantation, T cell activation was suppressed for a long time. The MSCs may be activated by inflammatory environment in the lung in the early stage and secrete a large amount of CCL2 to attract more CCR2-expressed T cells to infiltrate to lung tissue. At the same time, we found that the proportion of CCR2+CD4+ T cells in MSC-treated mice was always higher than that in IPS mice. The sustained increase in the proportion of CCR2+CD4+ T cells may be the effect of high concentration of CCL2 secreted by MSCs at early days and subsequently lung interstitial cells. Due to the immunoregulatory effect of CCR2+CD4+ T cells, the clinical symptoms, pathological manifestations and the survival of mice received MSCs prophylaxis were better than that of mice in the IPS group. Both CCR2 and CCL2 antagonists can significantly reversed the effect of MSCs in vivo, further indicating the pivotal role of CCR2-CCL2 axis in the prophylactic mechanism of MSCs on IPS.

In summary, this in vitro and in vivo study provides the first evidence of CCR2-CCL2 axis as a novel mechanism of MSCs modulating T cell function. MSCs alleviate IPS at least partially through driving T cells toward CCR2+CD4+ T cells polarization. CCR2-CCL2 axis and CCR2+CD4+ T cells might be the major molecular and cellular mechanisms for the effective prophylaxis and treatment of IPS by using MSCs. Our study provides theoretic foundation for the treatment of immune related diseases using MSCs, chemokine CCL2, or CCR2+CD4+ T cells.

## Conclusions

We demonstrated an important role of CCR2-CCL2 axis in modulating T cells function which is one of the mechanisms of the prophylactic effect of MSCs on IPS.

## Supplementary Information

**Additional file 1.** Supplementary figures.

## Data Availability

Data sharing is not applicable to this article as no datasets were generated or analyzed during the current study.

## Abbreviations

IPS: Idiopathic pneumonia syndrome
MSCs: Mesenchymal stem cells
allo-HSCT: Allogeneic hematopoietic stem cell transplantation),
aGVHD: Acute graft-versus-host disease
CCR2: Chemokine receptor 2
CCL2: Chemokine ligand 2
BALF: Bronchial alveolar lavage fluid
