# Supplementary material for: Mesenchymal stem cells alleviate idiopathic pneumonia syndrome by modulating T cell function through CCR2-CCL2 axis
Source: Stem Cell Res Ther. 2021 Jul 2;12:378. doi: 10.1186/s13287-021-02459-7 (PMC8254317; doi:10.1186/s13287-021-02459-7)
Supplement: Supplementary file 1 — Additional file 1. Supplementary figures. [file 13287_2021_2459_MOESM1_ESM.docx]

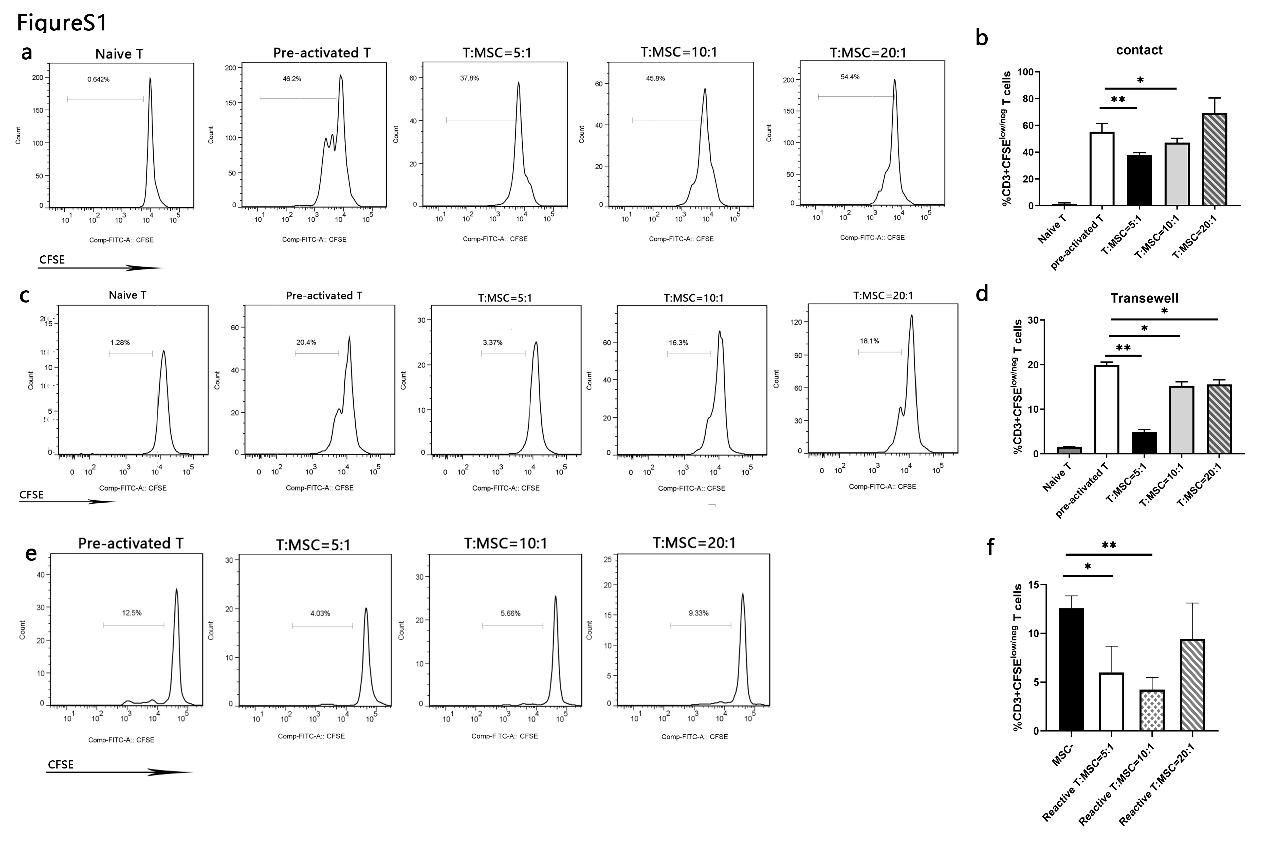


Figure legend

MSC inhibited the proliferation of T cells in the **(a-b)** contact culture system and in the **(c-d)** Transwell system after 48h co-culture. **(e-f)** MSC inhibited T cell proliferation in MLR.


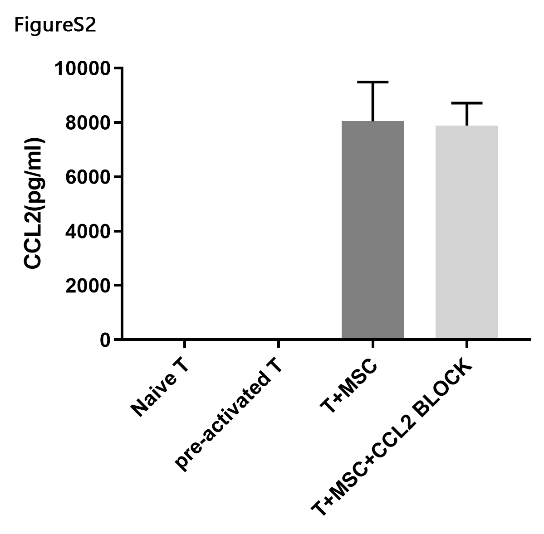


Figure legend

The addition of CCl2 antagonist to the system of co-culture of MSC and T cells can inhibit the activation and proliferation of T cells by MSC, but does not affect the secretion of CCl2 by MSC.


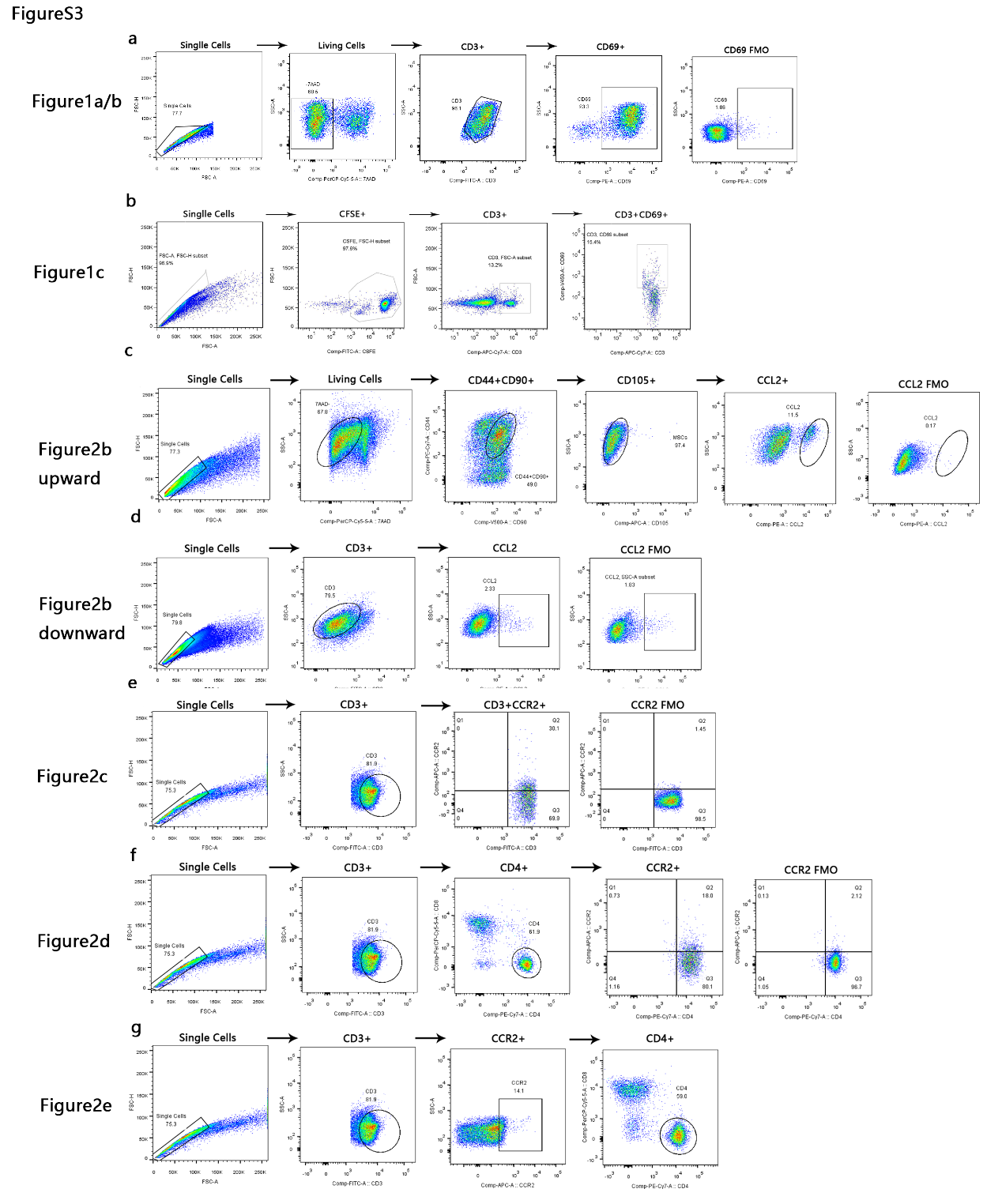


Figure legend

Gating strategy of **(a)** Figure1a and Figure1b **(b)** Figure1c **(c)** Figure2b upward **(d)** Figure2b downward **(e)** Figure2c **(f)** Figure2d and **(g)** Figure2e.


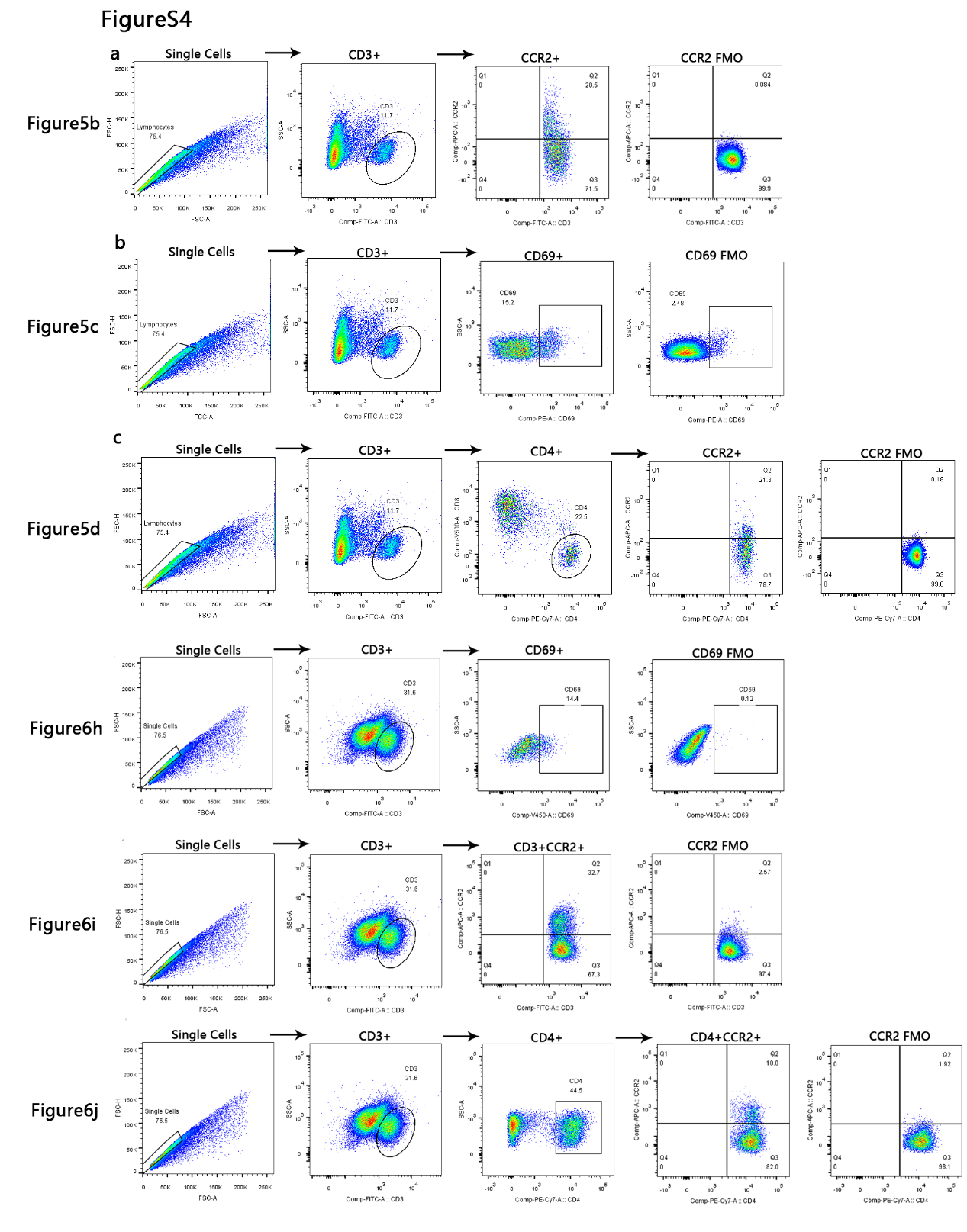


Figure legend

Gating strategy of **(a)** Figure5b **(b)** Figure5c **(c)** Figure5d **(d)** Figure6h **(e)** Figure6i and **(f)** Figure6j.


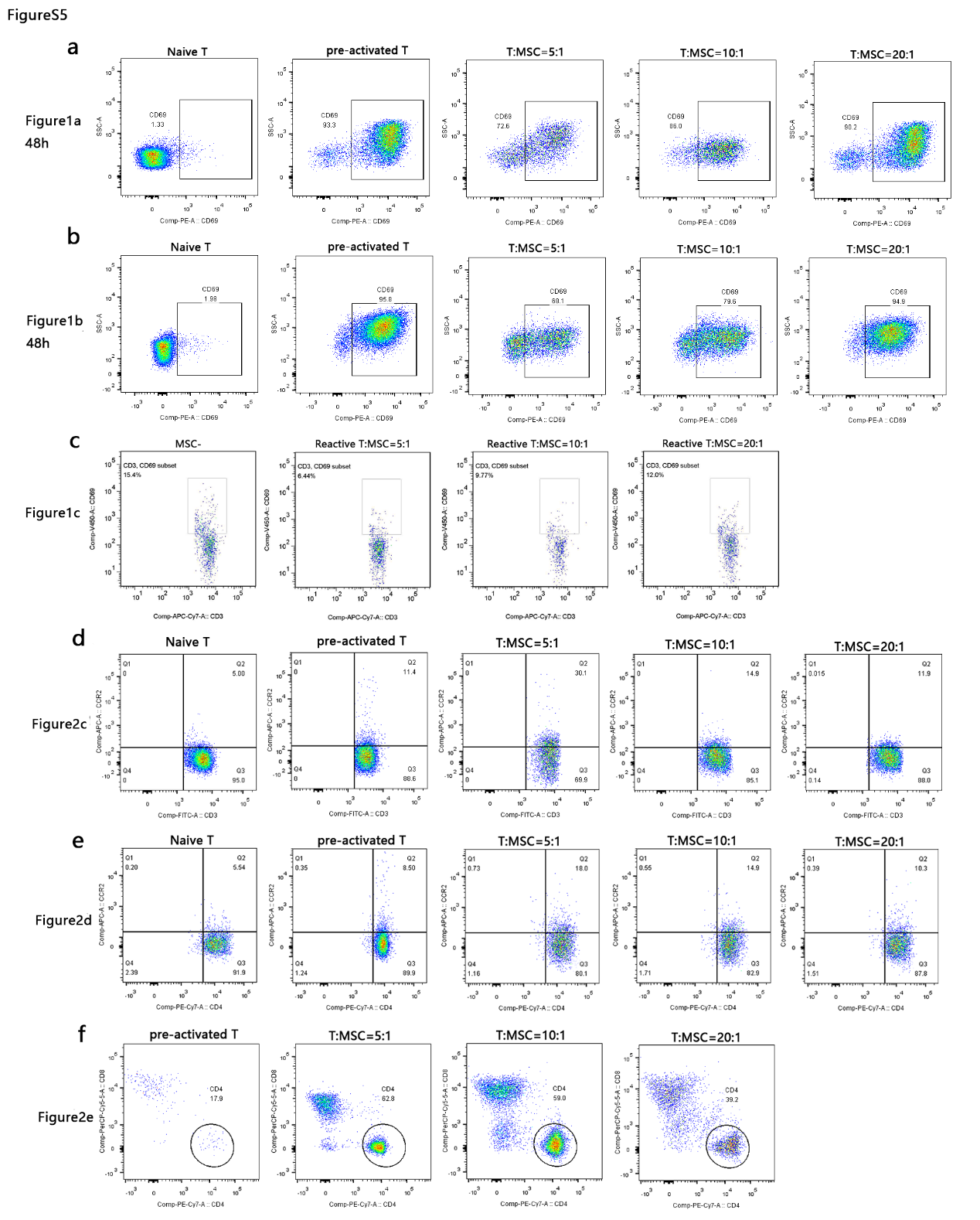


Figure legend

Representative cytometry data for **(a)** the 48h results of Figure 1a **(b)** Figure 1b **(c)** Figure1c **(d)** Figure2c **(e)** Figure2d **(d)** Figure2e.


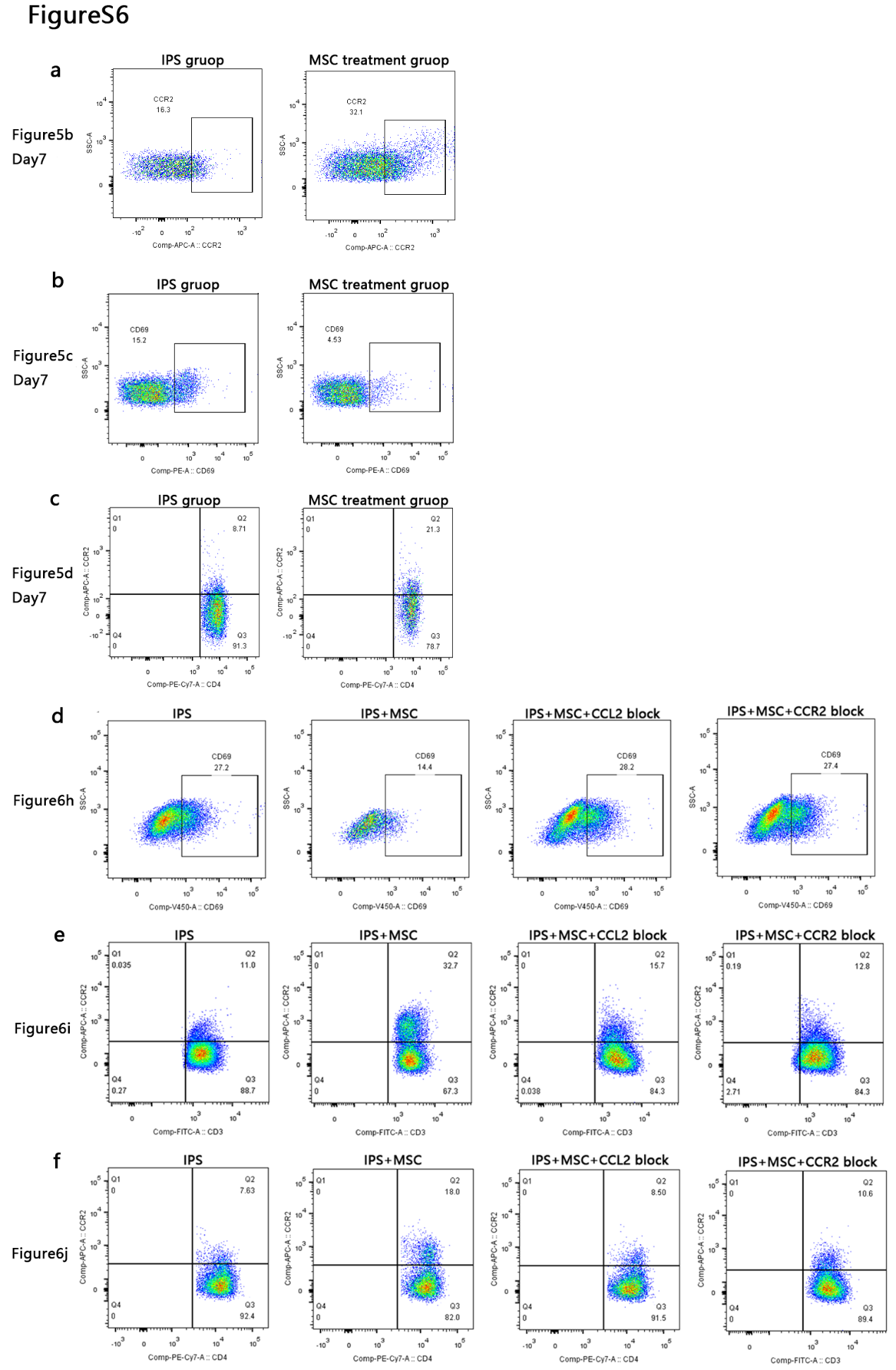


Figure legend

Representative cytometry data for the day 7 results of **(a)** Figure 5b **(b)** Figure 5c and **(c)** Figure5d**.** Representative cytometry data of **(d)** Figure 6h **(e)** Figure 6i and **(f)** Figure6j**.**
